# Effects of *Trichoderma* treatments on the phenolic and sensory quality of Aglianico grapes and wine

**DOI:** 10.1002/jsfa.70139

**Published:** 2025-08-29

**Authors:** Maria Tiziana Lisanti, Sheridan L. Woo, Angelita Gambuti, Roberta Marra, Giada d'Errico, Francesco Vinale, Gelsomina Manganiello, Luigi Moio, Nadia Lombardi

**Affiliations:** ^1^ Department of Agricultural Sciences University of Naples Federico II Portici Italy; ^2^ Department of Pharmacy University of Naples Federico II Naples Italy; ^3^ National Research Council Institute for Sustainable Plant Protection Portici Italy; ^4^ Center for Studies on Bioinspired Agro‐Environmental Technology (BAT Center), University of Naples Federico II Naples Italy; ^5^ Department of Veterinary Medicine and Animal Productions University of Naples Federico II Naples Italy

**Keywords:** bioactive fungal metabolites, red wine, polyphenols, sensory analysis, sustainable viticulture

## Abstract

**BACKGROUND:**

As environmental awareness grows, interest in sustainable agriculture is increasing. A promising alternative is the use of plant‐beneficial microorganisms such as *Trichoderma* spp., which suppress pathogens, promote growth and enhance productivity. In viticulture, *Trichoderma* species have been studied mainly for pathogen control, but their impact on wine composition and quality remains underexplored. The present study evaluates the effects of *Trichoderma afroharzianum* T22 and its metabolite, 6‐pentyl‐α‐pyrone (6PP) on *Vitis vinifera* cv. Aglianico over 2 years. Biometric parameters (grape yield per vine, 100‐berry weight), basic chemical parameters (soluble solids, pH, titratable acidity) and polyphenols (anthocyanins, high‐molecular‐weight tannins, vanillin‐reactive flavans) were analyzed in grapes. The resulting wines were assessed for phenolic composition and sensory attributes.

**RESULTS:**

Treatments increased anthocyanin content in both grapes and wine, at the same time as reducing low‐molecular‐weight tannins in grape skins, potentially decreasing bitterness. Despite an increase in high‐molecular‐weight tannins, no significant differences in astringency perception were detected. The wines from treated vines showed enhanced odor complexity, with stronger floral, tobacco and black pepper notes, likely a result of the increased terpenic volatile compounds. The effect of *T. Afroharzianum* T22 spores was more pronounced than that of 6PP.

**CONCLUSION:**

This study highlights the potential of *Trichoderma*‐based treatments as eco‐friendly alternatives to synthetic chemicals in viticulture. Beyond disease control, *Trichoderma* spp. and their metabolites may positively influence grape composition and wine quality, contributing to a more sustainable viticultural model. Further research is needed to better understand their effects on grapevine physiology and metabolism. © 2025 The Author(s). *Journal of the Science of Food and Agriculture* published by John Wiley & Sons Ltd on behalf of Society of Chemical Industry.

## INTRODUCTION

Viticulture and wine production are crucial to many economies but face challenges from pests and diseases exacerbated by climate change, which require effective management. Conventional viticulture relies heavily on chemical pesticides, which, although effective, accumulate in soils, harm non‐target organisms, promote resistance and pose potential risks to wine safety and quality.^1^, ^2^ With increasing environmental awareness, there is growing interest in sustainable agricultural methods. The European Union's Green Deal and Farm‐to‐Fork Strategy aims to convert 25% of farmland to organic practices by 2030, highlighting the need for eco‐friendly alternatives to synthetic chemicals.

A promising alternative for sustainable agriculture is the use of plant‐beneficial microorganisms such as *Trichoderma* spp., which can effectively suppress pathogens, stimulate plant growth and increase crop yields. These fungi are incorporated into over 250 commercial formulations, functioning as biofungicides, biopesticides and biostimulants. Both the direct application of *Trichoderma* strains and their purified bioactive metabolites have been shown to enhance plant resistance, improve physiological responses and increase antioxidant levels across a range of crop species.^3^, ^4^, ^5^, ^6^

Numerous studies have reported beneficial effects of *Trichoderma* application in many crops. In lettuce and rocket, they stimulated growth and enhanced native soil nitrogen uptake,^7^ whereas, in soybean and lentils, they increased key nutrient content, such as fatty acids, lipids and iron.^8^, ^9^ Field studies on strawberries showed improved yield, antioxidant levels and modulation of proteins involved in multiple functional categories.^10^, ^11^ Moreover, the use of *Trichoderma* demonstrated significant potential to enhance both the yield and nutrient content of tomatoes cultivated in greenhouse conditions.^12^, ^13^

In viticulture, *Trichoderma* spp. are commonly explored for pathogen control.^14^, ^15^ Products based on *Trichoderma* have proven effective as biocontrol agents against grapevine trunk diseases, with positive results observed both under controlled conditions and in field trials.^16^, ^17^ *Trichoderma afroharzianum* and *Trichoderma atroviride* are currently among the most widely used biocontrol agents in grapevine nurseries and vineyards, and their efficacy also extends to the control of foliar and bunch diseases.^18^, ^19^ However, the biostimulant potential of *Trichoderma* on grapevine growth and productivity remains less explored. Some evidence suggests beneficial effects on root development,^16^ whereas treatments with *Trichoderma harzianum* have also been associated with increased yield and improvements in quality parameters such as polyphenol content and antioxidant activity.^4^

Phenolic compounds in grapes, including anthocyanins, flavanols and tannins, are essential for plant defense and red wine quality, affecting color, astringency, bitterness and shelf life.^20^ This study evaluates the effects of *T. afroharzianum* T22 and its metabolite, 6‐pentyl‐α‐pyrone (6PP), on *Vitis vinifera* cv. Aglianico under vineyard conditions. Treatments were followed by analysis of biometric and chemical parameters, focusing on phenolic content. Phenolic compounds were extracted in a model solution from grape skins and seeds and assessed for their contribution to wine quality. Laboratory‐scale vinifications were performed, and the resulting wines were analyzed for phenolic composition and sensory attributes, offering a ‘vineyard‐to‐wine’ approach to studying the impact of *Trichoderma*.

## MATERIALS AND METHODS

### Fungal material and bioactive metabolite

In the experiments, biofungicide Trianum P® was used (Koppert Biological Systems, Berkel en Rodenrijs, The Netherlands), containing *T. afroharzianum* (ex‐*T. harzianum*) Rifai strain T22, a registered biological control agent.^10^, ^21^ Plants were watered with a conidia suspension at 1.0 × 10^7^ conidia mL^–1^. The *Trichoderma* metabolite 6‐pentyl‐α‐pyrone (6PP; Sigma‐Aldrich, Merck KGaA, Darmstadt, Germany) was resuspended in ethyl acetate (0.1% v/v), then diluted in distilled water, left in agitation to evaporate the ethyl acetate under a cabinet flow hood, and diluted in distilled water to a final concentration of 10^−6^ m for the treatments.

### Field treatments

The field experiment was conducted in 2016–2017 at Luigi Maffini Winery, Giungano (SA), Italy, in a 10‐year‐old *V. vinifera* cv. Aglianico vineyard (rootstock K5BB) with vine spacing of 0.90 m and row spacing of 2.30 m. The vineyard was managed using an espalier training system with single‐Guyot pruning. The soil was managed through mechanical tillage using a ripper (to a depth of 30 cm). A controlled cover cropping strategy was applied by mowing the naturally occurring (spontaneous) herbaceous species present in the vineyard.

A randomized block design with two blocks in different vineyard zones was used, each containing two biological treatments and a water control, each replicated twice. Each plot had seven treated plants in a single row, separated by seven untreated plants. Treatments included: (i) *T. afroharzianum* T22 spore suspension (10^7^ spores mL^–1^) applied by soil watering at 1 L per vine to the collar base, targeting the area closest to the root zone (Tr‐sp); (ii) 6PP solution (1 μm) applied by foliar spray at 100 mL per plant onto the upper and lower surfaces of the leaves until runoff (6PP); and (iii) water control (Control). All the applications were performed in the early morning (between 07.00 h and 08.00 h) to minimize evaporation and maximize absorption under lower light and temperature conditions, which can influence the effectiveness of foliar treatments. Treatments started in April at budbreak and continued monthly until August for a total of five applications. Disease symptoms of downy mildew, powdery mildew, and grey mold were monitored through regular visual inspections of the plants. Observations were performed by examining the surfaces of leaves, stems, and any visible fruits. Plant protection treatments applied during the experiment included the use of sulfur (both wettable and dust commercial formulations) and copper‐based products (copper oxychloride and copper hydroxide), with a maximum application rate of 3.5 kg of metallic copper per hectare per year. To minimize potential negative interactions between plant protection treatments and *Trichoderma*, products were chosen for their compatibility with *Trichoderma*, and applications were timed to prevent overlap with chemical treatments. During the trial period (from budbreak to harvest), agronomic operations included two shoot‐thinning interventions following winter pruning, along with several pinching‐off the shoot tips and, when necessary, leaf removal.

Grapes were hand‐harvested at technological maturity (stable sugar concentration over 1 week).^22^ The harvest dates were uniform across all treatments, including the control, and were carried out on 6 September 2016 and 4 September 2017. No significant differences in ripening dynamics or harvest timing were observed, suggesting that the treatments did not affect the phenological development of the grapes in terms of harvest readiness.

Grapes were then transported to the Division of Grape and Wine Sciences of the University of Naples Federico II for further analysis and vinification. Grapes from the two blocks were combined each year to create homogeneous treatment samples for laboratory experiments.

### Biometric parameters and basic chemical analyses of grapes

Grape yield per vine and 100‐berry weight were recorded for each treatment. Basic chemical parameters (soluble solids, pH and titratable acidity) were measured following the OIV methods of analysis,^23^ with all analyses in duplicate.

### Seeds and skins extractions

Extractions were performed on 100 berries per treatment, with two replicates. Berries were weighed, then seeds and skins were manually separated, weighed, and placed in glass bottles with 125 mL of model wine solution (ethanol 12% v/v, tartaric acid 5 g L^–1^, pH 3.2). Extractions occurred at 30 °C for 7 days with stirring twice daily. The final solutions were paper filtered and analyzed.

### Experimental vinification

Small‐scale vinifications followed a red winemaking protocol. One kilogram of grapes was de‐stemmed and crushed, then treated with potassium metabisulfite (60 mg L^–1^) and *Saccharomyces cerevisiae* yeast (MYCOFERM CRU 0.5, EVER, 0.45 g/L^−1^) was inoculated. Fermentation, conducted at 26 °C, included twice‐daily punch‐downs and analytical monitoring. After 12 days, when sugar content fell below 3 g L^–1^, wines were racked, sulfited (60 mg L^–1^), cold stabilized, filtered and bottled in 250‐mL bottles. Two vinification replicates were performed per treatment.

### Polyphenol analysis of grapes and wines

Anthocyanins and high‐molecular‐weight tannins were analyzed following Harbertson *et al* ^24^ Vanillin‐reactive flavans were measured following di Stefano *et al*.,^25^ with solvent use reduced as in Gambuti *et al*.^26^ Each extraction (skins and seeds) and vinification replicate was analyzed in duplicate. A Jenway 7305 spectrophotometer (Fisher Scientific Italia, Segrate, Italy) was used.

### Sensory analysis of wines

Wines from the 2017 experiment underwent sensory analysis by eight trained judges (five females, three males, aged 22–49 years), recruited from the staff and the students of the Division of Vine and Wine Sciences of the University of Naples Federico II. The study followed ethical standards set by the institutional and national research committees and the 1964 Declaration of Helsinki, including amendments. Participants' rights and privacy were protected, with no personal data disclosed without their consent, and data were collected anonymously. Participation was entirely voluntary and free from coercion. Before taking part in the study, participants were required to sign an informed consent form, which provided comprehensive information about the voluntary nature of the study (including the right to withdraw at any time), the research objectives, study requirements, potential risks, and the agreement to evaluate reference solutions and wines, without ingestion.

Judges were trained to evaluate taste, astringency, and odor attributes of red wine. Odor descriptors were generated, refined by consensus, and validated against previous studies on Aglianico wines.^27^, ^28^ Odor complexity, defined as ‘the richness of distinguishable odors’, was also assessed.

Taste and mouthfeel attributes included sweetness, acidity, bitterness and astringency, rated on a numerical nine‐point numerical scale, labeled as follows: 1 = very weak, 2 = weak, 3 = medium, 4 = strong, 5 = very strong. Intermediate half‐point values (e.g. 1.5, 2.5) were allowed, resulting in a total of nine possible evaluation points. Non‐rated descriptors were scored 0 by the experimenter. Sensory analyses were conducted in individual booths. Samples (30 mL) were served at 20 ± 2 °C in black tulip‐shaped glasses coded with three‐digit random numbers, with randomized presentation order. Judges rinsed with water between samples. Wines from two vinification replicates per treatment were combined and analyzed in duplicate.

### Statistical analysis

Given the small sample sizes and the non‐normal distribution or ordinal nature of several variables (e.g. sensory data), the non‐parametric Kruskal–Wallis test was used to detect differences among samples. When the Kruskal–Wallis test indicated significance (*P* < 0.05), the Conover–Iman post‐hoc test was applied for pairwise comparisons. *P* < 0.05 was considered statistically significant. Normal distribution was checked by Levene's test. All statistical analyses were performed using XLSTAT software (Addinsoft, Paris, France).

## RESULTS AND DISCUSSION

In 2017, higher solar radiation and temperatures were recorded compared to 2016 in the Campania region (weather station located at 40°28′24.71″N, 14°59′19.87″E; data not shown) (Regione Campania, 2016, 2017).^29^ These conditions caused increased abiotic stress, including higher radiation, air temperature and soil water deficits. No significant disease symptoms from foliar or grape cluster pathogens were observed in either year, likely as a result of the high temperatures and low humidity hindering pathogen establishment.

### Biometric parameters and basic chemical analyses of grapes

**Figure 1:**
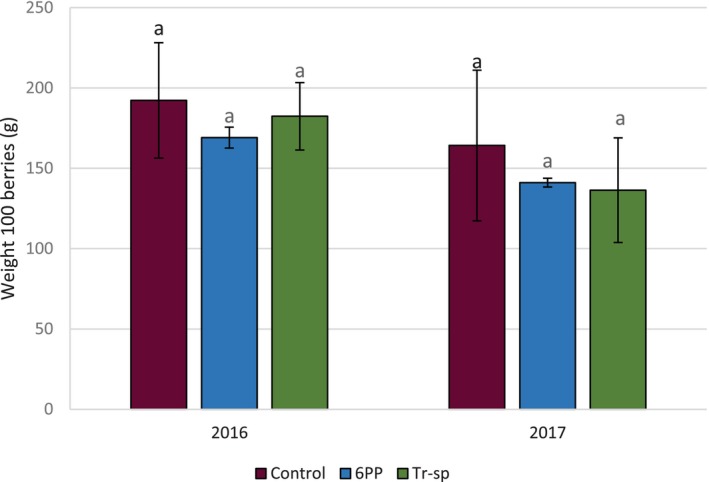
Weight of 100 Aglianico berries from vines treated with 6‐pentyl‐α‐pyrone applied as foliar spray (6PP) or *T. afroharzianum* strain T22 spores applied by watering (Tr‐sp) in 2016 and 2017. Control vines were treated with water. Similar letters within each year indicate no significant differences (*P* < 0.05).

The biometric parameters of grapes showed no significant differences in both years. The mean yield ranged from 1.0 to 1.1 kg per vine in 2016 and from 0.9 to 1.2 kg per vine in 2017, with no significant differences among treatments. The weight of 100 berries in 2016 ranged from 169.1 g to 192.3 g, whereas, in 2017, it was slightly lower, from 136.3 g to 164.2 g (Fig. 1). A similar trend was observed by Santos *et al*.,^30^ under abiotic stress conditions like those in 2017.

Among basic chemical parameters (Table 1), soluble solids content and pH were unaffected by the biological treatments in either year. In 2016, 6PP resulted in lower total acidity than the control, while in 2017, no significant differences were observed.

**Table 1:** Basic chemical parameters of Aglianico grapes from vines treated with 6‐pentyl‐α‐pyrone applied as foliar spray (6PP) or *T. afroharzianum* strain T22 spores applied by watering (Tr‐sp) in 2016 and 2017

	2016			2017		
	Soluble solids (°Brix)	Total acidity (g L^–1^ tartaric acid)	pH	Soluble solids (°Brix)	Total acidity (g L^–1^ tartaric acid)	pH
Control	22.1 ± 1.2 a	7.3 ± 0.3 b	2.98 ± 0.05 a	21.7 ± 0.3 a	6.5 ± 1.4 a	3.5 ± 0.10 a
6PP	21.7 ± 0.5 a	5.6 ± 0.7 a	2.97 ± 0.04 a	21.9 ± 0.8 a	6.7 ± 1.5 a	3.7 ± 0.04 a
Tr‐sp	23.0 ± 0.6 a	7.7 ± 0.7 b	2.97 ± 0.01 a	21.8 ± 0.3 a	6.8 ± 1.5 a	3.6 ± 0.07 a

*Note*: Control vines were treated with water. In each column, different lowercase letters indicate significant differences (*P* < 0.05).

### Polyphenol analysis of grapes and wines

#### Total anthocyanins

**Figure 2:**
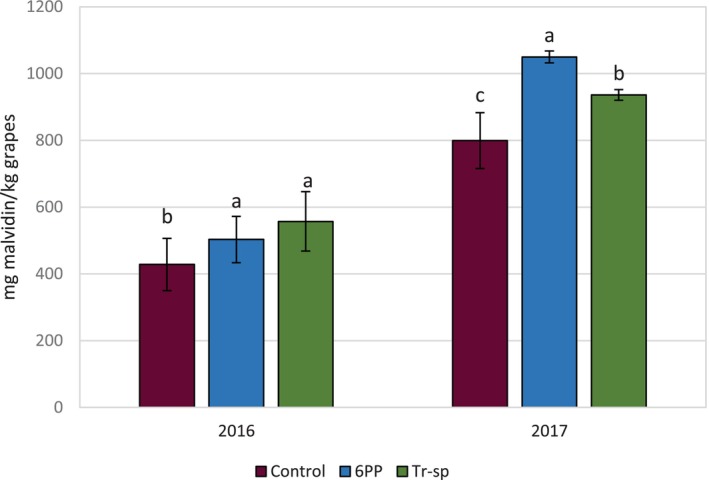
Total anthocyanin content (expressed as mg malvidin kg^–1^ grapes) from Aglianico skin extracts of grapes from vines treated with 6‐pentyl‐α‐pyrone applied as foliar spray (6PP) or *T. afroharzianum* strain T22 spores applied as watering (Tr‐sp) in 2016 and 2017. Control vines were treated with water. Different letters within each year indicate significant differences (*P* < 0.05).

In 2017, anthocyanin content from grape skins was nearly 50% higher than in 2016 (Fig. 2), likely as a result of a higher skin/pulp ratio and lower berry weight resulting from the warmer growing season. In 2016, both 6PP and *Trichoderma* treatments increased anthocyanin levels compared to the control, though they were not significantly different between them. In 2017, both treatments significantly boosted anthocyanin levels, with 6PP showing the greatest effect (Fig. 2). These results are consistent with a previous study in which treatments of vines with *T. harzianum* strain T22 spores and 6PP led to a higher polyphenol content in grapes.^4^

**Figure 3:**
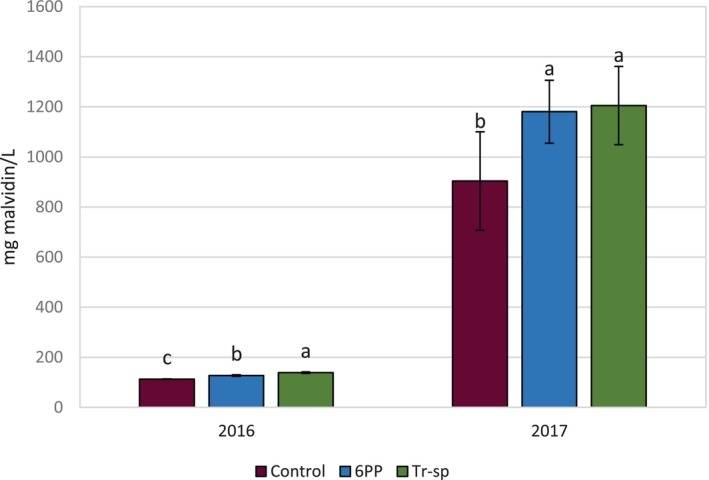
Total anthocyanin content (expressed as mg malvidin L^–1^) of wines from Aglianico grapes from vines treated with 6‐pentyl‐α‐pyrone applied as foliar spray (6PP) or *T. afroharzianum* strain T22 spores applied as watering (Tr‐sp) in 2016 and 2017. Control vines were treated with water. Different letters within each year indicate significant differences (*P* < 0.05).

The 2017 wines contained eight to nine times more anthocyanins than those from 2016 (Fig. 3). In both years, wine analysis showed higher anthocyanin content in treatments with 6PP or Tr‐sp compared to the control, mirroring results from 2016 and 2017 grape samples (Fig. 3). This suggests improved wine quality, as higher anthocyanin levels enhance color stability, oxidation protection^31^, ^32^ and potential health benefits.^33^

#### High‐molecular weight tannins

**Figure 4:**
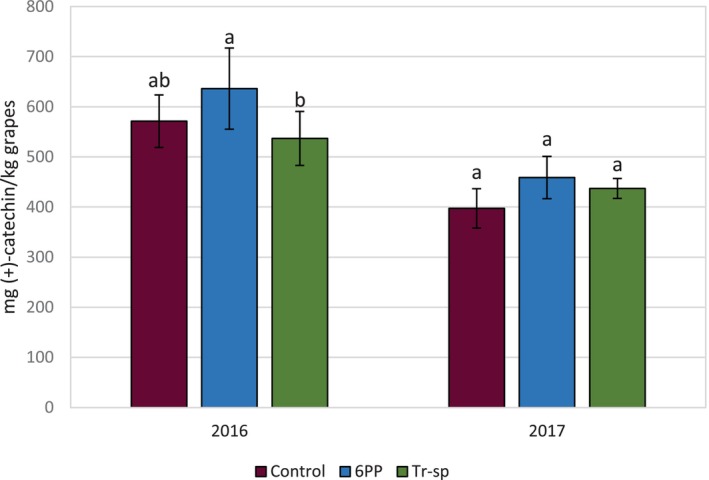
High‐molecular weight tannins (expressed as mg (+)‐catechin kg^–1^ grapes) from Aglianico skin extracts of grapes from vines treated with 6‐pentyl‐α‐pyrone applied as foliar spray (6PP) or *T. afroharzianum* strain T22 spores applied as watering (Tr‐sp) in 2016 and 2017. Control vines were treated with water. Different letters within each year indicate significant differences (*P* < 0.05).

**Figure 5:**
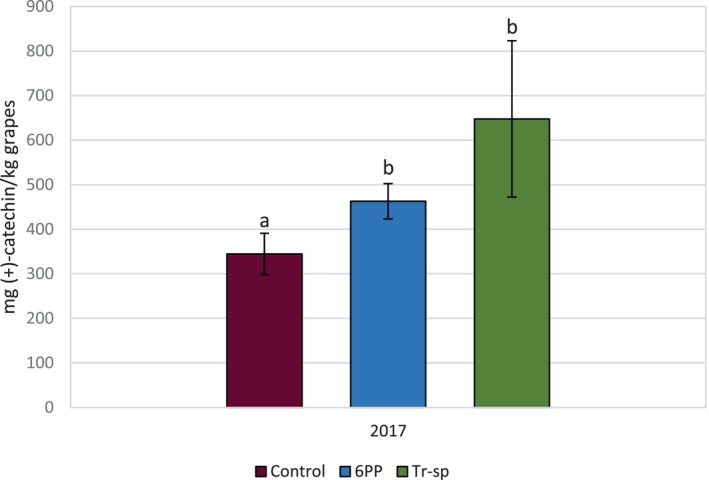
High‐molecular weight tannins (expressed as mg (+)‐catechin kg^–1^ grapes) from Aglianico seed extracts of grapes from vines treated with 6‐pentyl‐α‐pyrone applied as foliar spray (6PP) or *T. afroharzianum* strain T22 spores applied as watering (Tr‐sp) in 2017. Control vines were treated with water. Different letters indicate significant differences (*P* < 0.05).

High‐molecular weight (HMW) tannins were measured using the BSA assay^24^ to assess proanthocyanidins with a polymerization degree from trimers to octamers,^34^ correlating strongly with sensory astringency (*r* ^2^ = 0.82–0.90).^35^, ^36^, ^37^ In 2016, HMW tannin content in 6PP‐treated grape skins was significantly higher than in Tr‐sp, but both treatments were not significantly different from the control (Fig. 4). In 2017, no significant differences were observed, and HMW tannin levels were lower than in 2016 (Fig. 4). HMW tannins were not detected in 2016 seed samples, but, in 2017, significantly higher concentrations were found in both treated samples (6PP and Tr‐sp) compared to the control (Fig. 5). As the assay measures tannins reactive to BSA, precipitation increases with tannin polymerization size, ranging from trimers to octamers.^24^ Tannin size in 2016 may have differed, either being smaller or larger than detectable values. Year‐to‐year differences in tannin content and molecular size across red grape varieties have been reported^38^; however, the influence of environmental factors on the mean degree of polymerization remains unclear.^39^

The high temperatures in 2017 may have caused an interaction between abiotic stress and *Trichoderma*‐induced resistance, increasing HMW tannin accumulation in seeds. This suggests that, under climate change‐related stress, the vine's response to *Trichoderma* may differ from that under normal conditions. Further studies under extreme climate conditions are needed.

**Figure 6:**
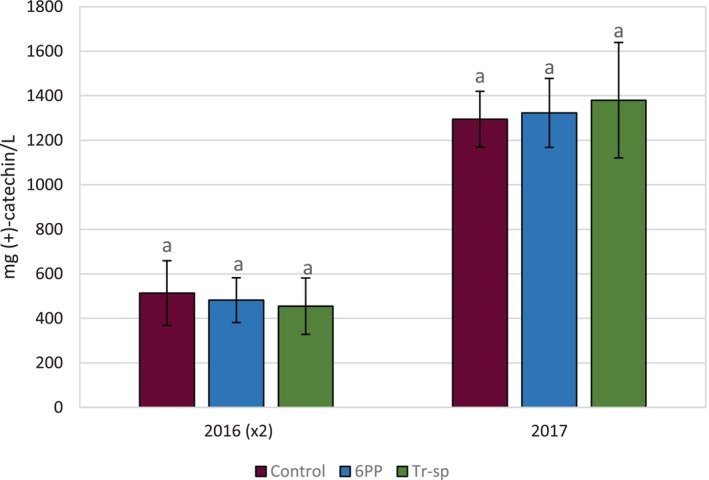
High‐molecular weight tannins (expressed as mg (+)‐catechin L^–1^) in wines from Aglianico grapes from vines treated with 6‐pentyl‐α‐pyrone applied ad foliar spraying (6PP) or *T. afroharzianum* strain T22 spores applied as watering (Tr‐sp) in 2016 and 2017. Control vines were treated with water. (x2) = Concentrations values detected in 2016 are multiplied by 2 in the graph. Different letters within each year indicate significant differences (*P* < 0.05).

The concentration of HMW tannins in wines was 2.3–3‐fold higher in 2017 than in 2016 (Fig. 6), likely as a result of seed contributions, as these tannins were detected only in 2017 (Fig. 5). No significant differences were detected among samples in either year (Fig. 6).

#### Vanillin‐reactive flavans

**Figure 7:**
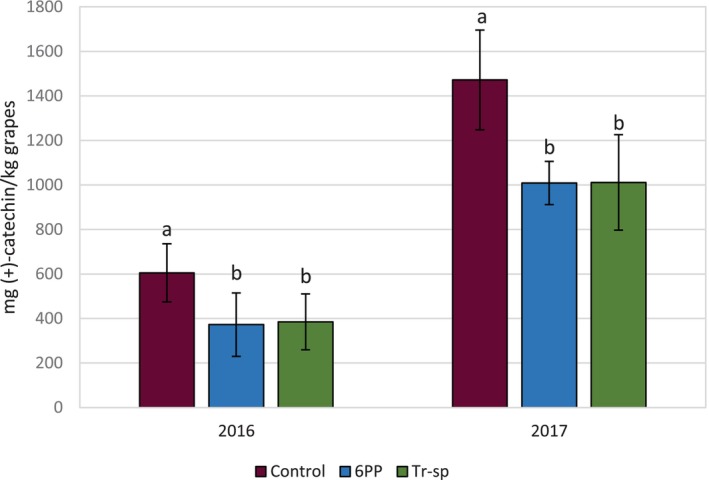
Vanillin‐reactive flavans (expressed as mg (+)‐catechin kg^–1^ grapes) from Aglianico skin extracts of grapes from vines treated with 6‐pentyl‐α‐pyrone applied as foliar spray (6PP) or *T. afroharzianum* strain T22 spores applied as watering (Tr‐sp) in 2016 and 2017. Control vines were treated with water. Different letters within each year indicate significant differences (*P* < 0.05).

Vanillin‐reactive flavans (VRF) estimate low‐molecular weight tannins, highlighting smaller, less polymerized structures.^40^ VRF levels in grape skin extracts were lower in 6PP‐ and *Trichoderma*‐treated plants compared to controls in both years (Fig. 7). In 2017, VRF content was more than double that observed in 2016, likely as a result of warmer conditions, which are linked to increased tannin synthesis in red grapes.^41^, ^42^ However, some studies report minimal temperature effects on flavanol synthesis.^43^ Discrepancies may be because of factors such as day–night temperature variations or also to the extraction methods. The higher VRF in 2017 could also reflect a higher skin/pulp ratio and lower berry weight. The lower VRF values in treated grape skins can be considered advantageous because wine bitterness is more closely associated with lower molecular weight compounds.^44^, ^45^

**Figure 8:**
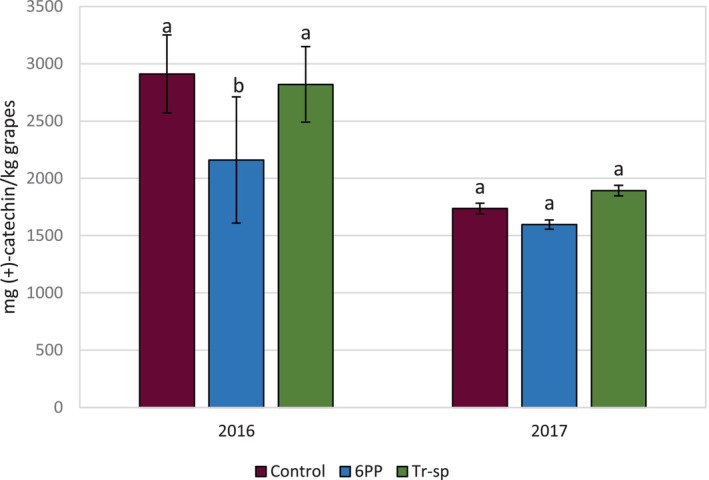
Vanillin‐reactive flavans (expressed as mg (+)‐catechin kg^–1^ grapes) from Aglianico seed extracts of grapes from vines treated with 6‐pentyl‐α‐pyrone applied as foliar spray (6PP) or *T. afroharzianum* strain T22 spores applied as watering (Tr‐sp) in 2016 and 2017. Control vines were treated with water. Different letters within each year indicate significant differences (*P* < 0.05).

VRF levels in seeds showed no significant differences across both years, except for a lower VRF content in 6PP‐treated samples in 2016, which was not observed in 2017 (Fig. 8). By contrast to the yearly fluctuations observed in grape skins, seed VRF production was 1.4‐ to 1.9‐fold higher in 2016 than in 2017 (Fig. 8). Overall, seed VRF levels surpassed grape skin levels, being 4.8‐ to 7.3‐fold higher in 2016 and 1.2‐ to 1.9‐fold higher in 2017 (Figs 7 and 8). The literature shows extractable tannins vary by grape variety, terroir, and vintage, with seed tannins reported to be up to 15 times higher than skin tannins at harvest.^39^ Additionally, grape skin tannins generally exhibit greater polymerization (2.1–85.7) than seed tannins (2.3–30.3),^39^ aligning with our findings.

**Figure 9:**
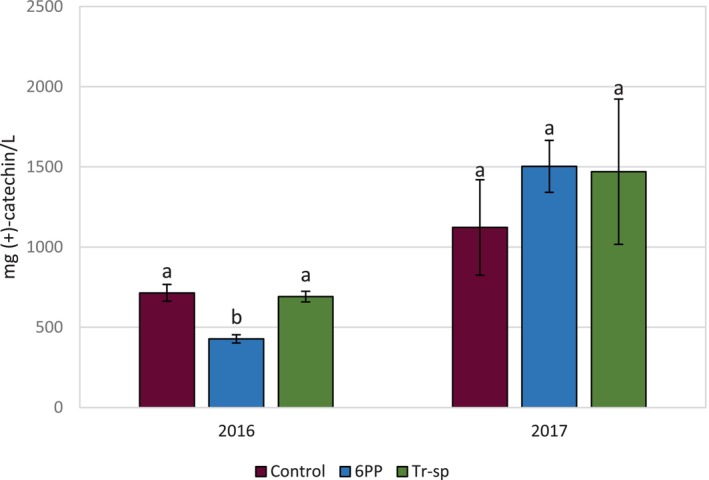
Vanillin‐reactive flavans (expressed as mg (+)‐catechin L^–1^) of wines from Aglianico grapes from vines treated with 6‐pentyl‐α‐pyrone applied as foliar spray (6PP) or *T. afroharzianum* strain T22 spores applied as watering (Tr‐sp) in 2016 and 2017. Control vines were treated with water. Different letters within each year indicate significant differences (*P* < 0.05).

VRF concentrations in experimental wines were similar to those in grape skins but lower than those in seeds. Wine analyses showed a strong influence of the production year, with higher tannin levels in 2017, reflecting trends in grape skins (Fig. 9). The 6PP and *Trichoderma* treatments showed no significant effects compared to the water control in 2017 (Fig. 9). In 2016, the 6PP treatment reduced VRF levels, similar to what was observed in seed extracts but at about one‐fifth of the concentration (Fig. 8).

Further controlled experiments could clarify whether *T. afroharzianum* and 6PP field treatments enhance anthocyanin biosynthesis at the same time as reducing the accumulation of flavanols and condensed tannins.

### Sensory analysis

**Figure 10:**
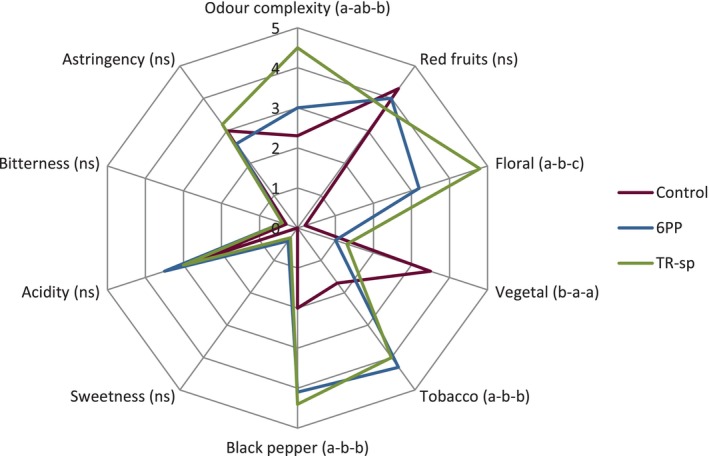
Sensory profiles of wines from Aglianico grapes from vines treated with 6‐pentyl‐α‐pyrone applied as foliar spray (6PP) or *T. afroharzianum* strain T22 spores applied as watering (Tr‐sp) in 2017. Control vines were treated with water. For each descriptor, different letters in brackets indicate significant differences among the samples (*P* < 0.05): the letter on the left refers to Control, the middle letter refers to 6PP, and the right letter refers to Tr‐sp; ns= not significant.

A sensory analysis of 2017 wines from grapevines treated with 6PP and TR‐sp assessed taste and mouthfeel (sweetness, acidity, bitterness, astringency) and odor attributes (red fruits, floral, vegetal, tobacco, black pepper). Odor complexity, defined as the ‘richness of distinguishable odors’, was rated higher in wines from treated vines, with TR‐sp samples showing notable enhancement (Fig. 10). This is a highly positive result because complexity is a key valued sensory characteristic in wine.^46^ Floral, tobacco and black pepper odors were stronger in wines from treated grapes, whereas red fruit showed no differences and vegetal odor was weaker compared to controls. Notably, floral odor intensity was 0.2 in controls but 3.2 (6PP) and 4.8 (TR‐sp) on a scale of 0–5 (Fig. 10). Floral, tobacco and black pepper odors in wine are linked to volatile compounds from grapes, either free or as precursors.^47^, ^48^, ^49^ Floral terpenes such as linalool, geraniol and nerol contribute to aroma and are detectable in Aglianico wine.^27^, ^28^ *Trichoderma* treatments in tomatoes activate terpenoid biosynthesis genes.^50^ A similar effect on gene expression or metabolic changes induced by *Trichoderma* may occur in grapevines. Further studies are needed to understand whether and how these eco‐sustainable field treatments enhance terpene biosynthesis in grapes, leading consequent sensory effects. The differences observed in individual odor descriptors are likely to enhance overall odor complexity, particularly in TR‐sp wine (Fig. 10). Lower vegetal odors, associated with a ‘green’ character and dry astringency, are considered positive.^51^ This green character has a negative connotation among wine experts.^51^ Enhanced odor complexity and reduced ‘green’ notes likely improved sensory quality. Future studies should explore whether sensory differences result from changes in volatile compounds and/or the non‐volatile matrix, which can affect wine aroma release, particularly the phenolic fraction.^52^

## CONCLUSIONS

For the first time, the present study evaluated the impact of biological treatments using fungal antagonist *T. afroharzianum* T22 and microbial metabolite 6PP on grape and wine quality, from vineyard to wine. The treatments increased anthocyanin content in both grapes and wine, while reducing low‐molecular weight tannins in grape skins, potentially lowering bitterness. Although high‐molecular weight tannins increased, no significant differences in perceived astringency were found. The treatments enhanced the wine's odor complexity, with stronger floral, tobacco and black pepper notes, likely due to terpenic volatile compounds. The findings of this study are expected to provide valuable insights into the potential of *Trichoderma* treatments as a sustainable alternative to synthetic chemical inputs in viticulture. Beyond their well‐documented role in pathogen control, *Trichoderma* spp. and their bioactive metabolites have demonstrated the ability to enhance grape composition and positively influence wine quality. This highlights their promise as an eco‐compatible strategy for improving both the sustainability and the enological value of vineyard production. This preliminary study provides useful insights for future investigations and highlights the potential of *Trichoderma* as a beneficial tool in sustainable viticulture.

Nonetheless, further investigations are required to elucidate the underlying mechanisms driving these effects on grapevine physiology.

## Data Availability

The data that support the findings of this study are available on request from the corresponding author. The data are not publicly available due to privacy or ethical restrictions.
